# Germplasm dynamics: the role of ecotypic diversity in shaping the patterns of genetic variation in *Lolium perenne*

**DOI:** 10.1038/srep22603

**Published:** 2016-03-03

**Authors:** T. Blackmore, D. Thorogood, L. Skøt, R. McMahon, W. Powell, M. Hegarty

**Affiliations:** 1 Institute of Biological, Environmental and Rural Sciences, Aberystwyth University, Gogerddan, Aberystwyth, Ceredigion, Wales. SY23 3EE.

## Abstract

Perennial ryegrass (*Lolium perenne*) is the most widely grown temperate grass species globally. Intensive plant breeding in ryegrass compared to many other crops species is a relatively recent exercise (last 100 years) and provides an interesting experimental system to trace the extent, impact and trajectory of undomesticated ecotypic variation represented in modern ryegrass cultivars. To explore germplasm dynamics in *Lolium perenne*, 2199 SNPs were genotyped in 716 ecotypes sampled from 90 European locations together with 249 cultivars representing 33 forage/amenity accessions. In addition three pseudo-cross mapping populations (450 individual recombinants) were genotyped to create a consensus genetic linkage map. Multivariate analyses revealed strong differentiation between cultivars with a small proportion of the ecotypic variation captured in improved cultivars. Ryegrass cultivars generated as part of a recurrent selection programme (RSP) are strongly associated with a small number of geographically localised Italian ecotypes which were among the founders of the RSP. Changes in haplotype frequency revealed signatures of selection in genes putatively involved in water-soluble carbohydrate (WSC) accumulation (a trait selected in the RSP). Retrospective analysis of germplasm in breeding programmes (germplasm dynamics) provides an experimental framework for the identification of candidate genes for novel traits such as WSC accumulation in ryegrass.

Plant species encounter a diverse set of selective pressures as they spread into new ecosystems, leading to changes in disease resistance, growth habit, production or partitioning of metabolites and gross morphology. Ecotypic material thus represents an important source of adaptive genetic variation which can be maintained by gene banks for use in conservation efforts or plant breeding. With the availability of high-density genotyping systems, this variation can be studied on a genome wide basis– both for understanding the genes which underpin adaptive traits, and also to facilitate their incorporation into plant breeding programmes for crop species^1^,^2^. These natural accessions can provide a source of useful traits which may have been lost to genetic erosion during the domestication process, and which can be reintroduced via introgression. This approach has been used to great success in long-domesticated species, such as rice, by breeding with wild relatives^2^. In more recently-domesticated species, there is the potential to investigate the impact of domestication on genetic diversity as well as identifying the genes underlying natural variation which has been incorporated into breeding programmes^3^. The forage grass *Lolium perenne*, has only recently (past 100 years) been subjected to selective breeding^4^ and provides an interesting experimental system to explore the dynamics and impact of domestication on genetic diversity in a recently evolved crop.

*Lolium perenne* (perennial ryegrass) is the most widespread grass species in temperate regions globally due to its rapid establishment, persistence and nutritional value to ruminants. It is outbreeding and exhibits extensive genetic variation for morphological growth characteristics^5^,^6^, extreme climate tolerance^7^,^8^, disease and pest resistance^9^,^10^ and soil mineral composition^11^,^12^. This variation is represented in ecotypes that are maintained in germplasm collections that can be characterised and utilised in breeding programmes to develop improved cultivars that are adapted to a range of biotic and abiotic stresses.

Due to the range of phenotypic traits exhibited by perennial ryegrass, it is commercially important as both a forage and amenity (recreational/turfgrass) species. Forage varieties of *L. perenne* are favoured due to their quick establishment, long growing season and high dry matter yield that is highly digestible to ruminants^13^,^14^,^15^. In contrast, amenity varieties are selected for the short growth habit, thicker sward from the increased tillering, persistence and resilience to close cutting^16^,^17^,^18^,^19^. Whilst the phenotypic differences between these functional groups are readily apparent, a genome wide analysis of the extent of differentiation and the biological pathways or genes under selection has not been previously reported.

The creation of a publically available genotyping array for *Lolium perenne* with over 2,000 validated markers provided the opportunity to interrogate germplasm in new ways^20^. The ability to genotype individuals with the same SNPs allows the comparison of large number of individuals, accessions, populations and meta-studies. Reproducible and comparable estimates of diversity and linkage disequilibrium will also allow the identification of genomic regions that are under selection and help guide the judicious choice of genetic diversity in breeding programmes. As part of ongoing studies to characterise ecotypic variation in perennial ryegrass, Blackmore *et al.*^20^ described and quantified genetic diversity detected in a sample of European ecotypes that reflected the geographic distribution and origin of the accessions. A large East-West cline was observed together with further genetic sub-structure that is related to latitudinal differences. Extensive genetic variation was detected in unimproved ecotypes that highlighted the opportunity to exploit this variation in ryegrass breeding.

In this study we exploit the Infinium array described previously^20^ to quantify the levels and patterns of genetic variation in commercial cultivars of forage and amenity grasses relative to that observed in ecotypes. We also show the capture of ecotypic variation from founders into a recurrent selection programme and observe the effects of selection on haplotypes of candidate genes for water-soluble carbohydrate (WSC) within this programme.

## Results

### Genetic Diversity

To evaluate levels of diversity and divergence, summary statistics were calculated within different groups of germplasm. Observed heterozygosity for each cultivar group was greater than the expected heterozygosity based on HW expectations, which was similar to the ecotype accessions (Table 1, Supplementary Tables S1 and S2). Consequently the fixation index was negative across all groups. The percentages of polymorphic loci were greatest in the OA and OF groups, and comparable to the ecotypes. This had only reduced to 76% in AF and 79% in ARSP. All cultivars showed similar levels of polymorphism, however, some had percentages of polymorphic loci below 70%, such as AF3 (Aurora), AF1 (S23-1970), OA8 (Bartwingo), AA1 (AberImp) and AA2 (AberSprite) (Supplementary table S2). Of which, Aurora and S23-1970 had less than 3 individuals genotyped.

An analysis of molecular variance (AMOVA) was conducted to examine the patterns of diversity between and within germplasm groups (Table 2). As expected for an outbreeding species most diversity was detected within populations although there were differences detected between the different germplasm groups. The lowest PhiPT value for the groups was seen in the AA group. AA shows little diversity, however, there are only 16 individuals representing 2 cultivars included in this group. OF has the second lowest value indicating less diversity between accessions than other groups. Despite the selective breeding program in Aberystwyth, AF showed the largest PhiPT value.

### Population structure in cultivars and ecotypes of L. perenne

**Figure 1:**
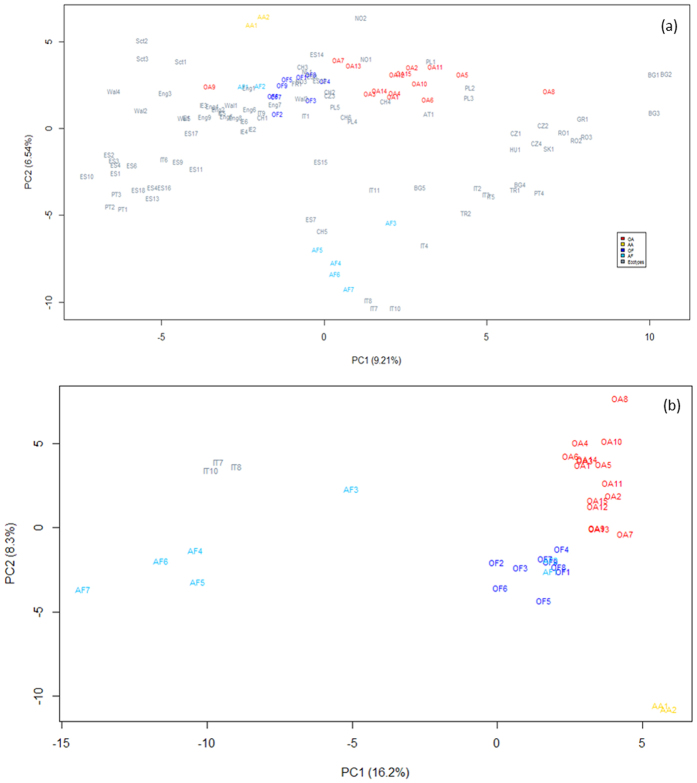
Principal component analysis of ecotypes and varieties. (**a**) Ecotypes (grey) identified by country ID (supplementary Table S1), amenity varieties- Aber amenities (AA; orange), other amenity varieties (OA; red); forage varieties–Aber forage (AF; light blue), other forage varieties (OF; blue) (supplementary Table S5). (**b**) Founder ecotypes used in ARSP (grey), amenity varieties- Aber amenities (AA; orange), other amenity varieties (OA; red); forage varieties–Aber forage (AF; light blue) with AF4-AF7 representing ARSP varieties, other forage varieties (OF; blue). Figure 1a modified from Blackmore *et al.*^20^.

In order to understand the distribution of genetic diversity contained in cultivars in comparison to the variation across European ecotypes, an unbiased principal component analysis (PCA) was performed on the allele frequency for each of the 2199 SNPs within each of the 90 sample locations (accession) in addition to the 33 cultivars (Fig. 1a). As previously reported^20^, the ecotypes are distributed in close alignment to their geographical origin. Together, the cultivars occupy a limited proportion of the genetic variation observed across Europe with most of the cultivars clustering in the central part of the PCA plot with forage varieties grouped apart from amenity, revealing that the genetic variation in *Lolium perenne* germplasm reflects their history of breeding for specific end user needs. Outliers from the central clusters were Aberystwyth’s amenity varieties, AberImp and AberSprite, and in the opposite direction on PC2 Aberystwyth’s Aurora and recurrent selection programme (ARSP) varieties (AF4-7; AberElan, AberAvon, AberDart and AberMagic). The ARSP shows progressive movement away from other forages towards 3 Italian ecotypes, IT7, 8 & 10. These ecotypes were, in fact, used in the original polycross with S23 (AF1 & 2) in the development of the ARSP (AF4-7). The breeding programme has focussed on recurrent selection for increased nutritional composition, in particular high WSC. Cultivar Aurora (AF3) is also descended from ecotypic material, CH6. A total of 21 tillers from accession CH6 were polycrossed and the progeny subjected to multiple rounds of crossing/selection for persistence, winter hardiness, early heading, yield and increased WSC, likely explaining the shift of AF3 away from its CH6 founder on Fig. 1a and towards the ARSP group, which shares these traits.

To focus further on the differences between the cultivars, PCA was performed on only the cultivar accessions along with the Italian ARSP founder ecotypes (Fig. 1b). This analysis clearly highlights the distinct genetic difference of the ARSP varieties in comparison to amenities and other forages. The top 20 markers contributing to the distribution of accessions along PC1 are listed at the top of Table 3. Although there are some uncharacterised or predicted proteins, there are 5 markers from 2 *L. perenne* contigs that have top BLAST hits to succinate dehydrogenase. In addition to which, markers associated to NADH dehydrogenase and hexokinase are also listed. PC2 significantly separates the Aberystwyth amenity varieties and the other commercial amenity varieties; it also discriminates between clusters of the forages and the amenities. The top 20 markers contributing the variation seen on PC2 are shown in the lower half of Table 3. Two markers which exhibit the greatest weight on PC2 are pheophorbide a chloroplast-like protein and aminoacylase-1-like protein. Two markers are associated with cell wall function; NAC secondary wall thickening promoting factor (Nst1) and cellulose synthase, are also identified in the top 20 markers (Table 3). In order to identify the markers and regions that segregate OF and OA, PCA was repeated for the cultivars (as in Fig. 1b), with the exception of the removal of the 2 AA accessions (Supplementary Fig. S1). The top 20 markers on PC2, which separated OF and OA are given in supplementary table S3. These markers have the greatest differentiation between the forage (OF) and amenity (OA) varieties. Pheophorbide a chloroplast like protein, again, had the greatest contribution to the segregation on PC2, indicating it differs between OF and OA. A number of other markers were identified including membrane proteins and stress related proteins, including sugar transporter erd6-like, alkaline/neutral invertase, snRK1-interacting protein I.

### Genomic regions under selection

In order to identify regions of the genome that have been under directional selection, a consensus linkage map was constructed from three distinct mapping populations that were genotyped with the Infinium array. Each mapping population generated its own linkage map with 1255, 1475 and 745 markers for the amenity x forage, AberMagic x Aurora and a F2 populations, respectively. Upon combining these three maps, 1386 markers of the 2199 validated markers were included in an integrated consensus map (supplementary Fig S2; Table 4). The markers were distributed evenly across all 7 linkage groups (LG), with the exception of LG5 where only 109 markers, against an average of 198 per linkage group, were assigned (Table 4). Across all linkage groups, 98% of the markers were less than 4cM apart.

**Figure 2:**
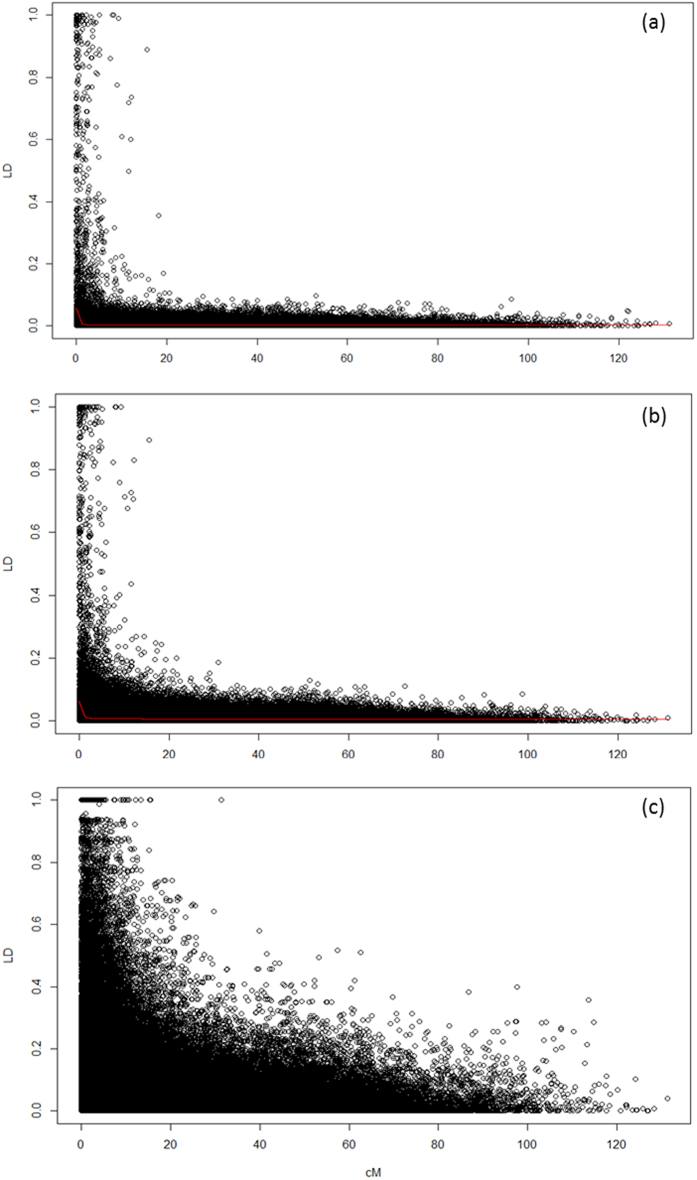
Linkage disequilibrium decay plot for ecotypes and varieties. LD breakdown across the genome in (**a**) natural ecotype populations; (**b**) commercial varieties, (**c**) Recurrent selection programme (ARSP). Red line denotes LOWESS curve.

Genetically mapped markers were used to determine the extent of linkage disequilibrium (LD) in natural *Lolium perenne* populations across Europe (Fig. 2a). LD across the genome (all linkage groups combined) was seen to rapidly breakdown in ecotypes (LOWESS curve did not extend up to r^2^ = 0.2). The genetic distance was slightly greater in commercial varieties (Fig. 2b). To focus further on the effect of a recurrent selection on a breeding programme, LD in ARSP was also plotted (Fig. 2c). ARSP included 4 forage cultivars (AberElan, AberAvon, AberDart, AberMagic) that are part of the recurrent selection programme with a focus on increasing WSC content. The greatest amount of LD was seen in this group, compared to varieties (Fig. 2b) and ecotypes (Fig. 2a). However, the LOWESS curve remained low and did not extend up to r^2^ = 0.2, highlighting that some regions have much greater levels of LD, but in general LD remains very low across the genome, as would be expected for an outbreeding, SI species. Examining LD for each LG found slightly elevated LD in LG1, 2, 4, 6 and 7.

**Figure 3:**
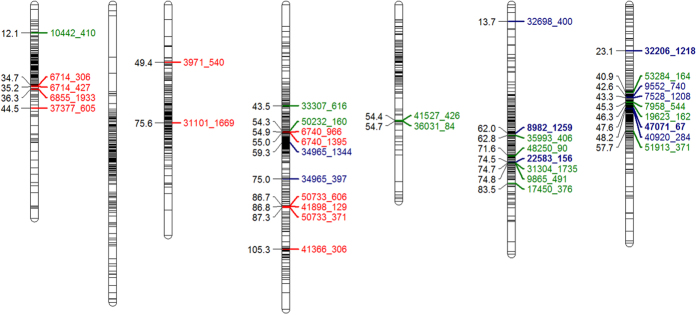
Consensus *Lolium perenne* genetic linkage map with 1386 SNP markers constructed from 3 mapping populations. Markers highlighted represent the mapped marker in top 20 loadings to i) PC1 (differentiating ARSP from OF & OA) (Table 3) (red); ii) PC2 (differentiating OA from AA) (Table 3) (blue); iii) PC2 without AA (differentiating OF from OA) (Supplementary Table 3) (green). Markers in blue bold are on Table 3 and Supplementary Table 3, differentiating OF and OA.

In order to genetically locate regions under selection for functional use (amenity or forage), the top 20 markers with the highest loading in the PCA plot (Fig. 1b) are identified on the consensus linkage map (Fig. 3). Strikingly, the markers contributing to the differentiation of ARSP from OA and OF are located on LG1, 3 and 4, with a cluster of markers on LG1 and 4. Markers with the greatest loading on PC2 are shown in tables 3 and supplementary Table 3 and are localised on LG6 and 7, identifying regions discriminating between amenity and forage groups.

**Figure 4:**
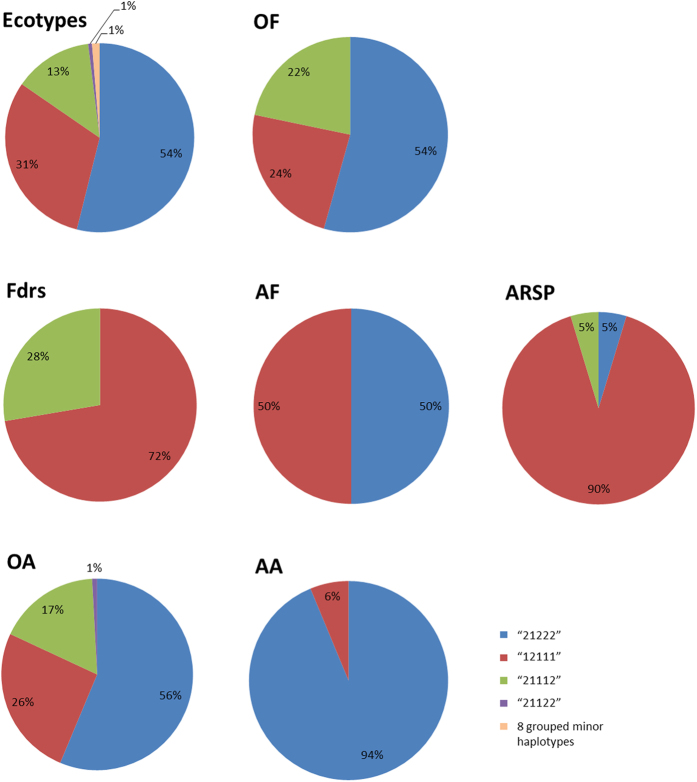
Haplotype frequency for succinate dehydrogenase across ecotypes and functional groups. Ecotypes–692 individuals spanning 87 geographic sample sites across Europe; Fdrs–ecotypes founders used in ARSP (24 individuals); AF1-3–AF1, AF2 & AF3 (13 individuals); ARSP–Recurrent selection programme (AF4-7) (32 individuals); OF–Other commercial forages (69 individuals); AA–Aberystwyth amenities (16 individuals); OA–other commercial amenities (119 individuals).

Of particular interest is the cluster of 3 SNPs at 86cM on LG4 from 2 different contigs that both BLAST to succinate dehydrogenase. These 2 contigs have a total of 5 markers on the array, with 2 currently unmapped. These 5 markers are all in the top 20 markers contributing to the loading on PC1, and therefore differentiating ARSP from OF and OA. SNP- based haplotype analysis was performed on these 5 markers for the ecotypes, Italian founders of the ARSP and other groups of interest (Fig. 4). This identified 12 haplotypes which were all present in the ecotypes (ecotypes + founders). Haplotype “12111” had a disproportionately high frequency in the founders, compared to the other ecotypes and other commercial varieties (OA and OF). The founders were used in conjunction with S23 (AF1-2) and recurrently selected in ARSP with an emphasis for increased WSC. Haplotype “12111” has been preferentially selected for in the ARSP, with the frequency of this haplotype increased to >90%, compared to ~31% in ecotypes (excluding the founders).

## Discussion

This study of natural variation in *L. perenne* is the largest, to date, in terms of individuals genotyped with a comprehensive, genomewide array of 2199 SNPs. It therefore provides insight into the extent and distribution of genetic diversity available in European ecotypes of *Lolium perenne* and how this variation has been incorporated and exploited in breeding programmes. As the most economically and agriculturally important temperate forage crop, understanding patterns and distribution of genetic variation will help breeders respond to the new challenges of sustainable intensification^21^.

The European ecotype sample previously described^20^ enabled a comparison with commercial forage and amenity synthetic varieties. Observed heterozygosity (Ho) was previously reported to be greater than expected heterozygosity (He) in all ecotypes^20^. The same observation was made in varieties (Supplementary Table S4). Explanation due to technical bias was excluded by comparison of observed heterozygote numbers in a biparental mapping population where both parents had been genotyped, and therefore the expected number of heterozygotes could be calculated. Variation of observed heterozygotes around the expected values was seen in the markers, but no deviation from 0 was found as an average across all the markers (Supplementary Table S4). Previously, when observed only in the ecotypes, this was proposed to result from polycrossing within accession to provide seed for germplasm bank storage at Aberystwyth, and the influence of self-incompatibility (SI) loci under these conditions^20^. Commercial cultivars are produced from a number of parental genotypes that may vary from 4 to hundreds^22^,^23^,^24^,^25^, however the observed excess of heterozygosity remains fairly constant across all accessions. As far as we know, this appears to be the first documented observation of this increase in heterozygosity in *Lolium perenne.* The effect at individual loci is non-significant and it is only due to the large number of markers and populations studied that the general effect is visible. We propose this effect arises from the action of SI and warrants further investigation.

Although the commercial varieties together capture a reasonable range of the genetic diversity observed across Europe, there was a surprising “geographic” divide seen between amenity and forage varieties genotypes. The amenities captured a greater expanse of the European ecotype diversity, with the forages forming a tight cluster on the PCA plot (Fig. 1a). This figure highlights ecotypic diversity that remains uncaptured by commercial breeding programmes. While this might be because these ecotypes lack key traits for amenity or forage use, this diversity may equally represent novel genetic resources for inclusion in present or future breeding programmes. LD analysis shows no significant increase in the magnitude of LD within cultivated ryegrass versus ecotypic material, again confirming that the genetic diversity within breeding programmes has not been unduly affected.

The ARSP genepool is differentiated from the other commercial forages, demonstrating the power of directional selection with a focus on increasing WSC. An individual from each of the ecotype accessions IT7, IT8 and IT10 were originally polycrossed with S23 and Ba9633 between 1978–1980. Recurrent selection of this population has led to a number of award-winning varieties, renowned for their increased WSC^14^,^26^. Genetic variation for WSC has previously been reported^27^ and has allowed the continued increase in WSC, from an average of 205 g/KG dry matter in AberDart to 237 g/KG in AberMagic from 7 cuts over 5 years. The benefits of increasing WSC have been shown to increase animal productivity and reduce nitrogen excretion^28^. PCA showing only the varieties highlights the progression of these cultivars away from the other forages on PC1 (Fig. 1b). The SNP markers contributing to the greatest difference in genetic variation show movement toward allele fixation in the ARSP, culminating in AberMagic (AF7). A number of interesting BLAST hits were found for the contigs in which the markers resided (Table 3). A common function of most of these candidates is their role in a stress response in plants, as well as energy metabolism. Five SNPs in 2 different contigs matched succinate dehydrogenase, which is associated with photosynthesis and stress responses in *Arabidopsis thaliana* and *Oryza sativa*^29^,^30^. NADH dehydrogenase has links with oxidative stress, increased growth and biomass^31^,^32^. Hexokinase 5 has been reported to be involved in fructan mobilisation in *Lolium perenne*^33^. Dihydrolipoyl dehydrogenase-like protein also has a role in photosynthesis and photorespiration^34^. Ryegrasses store their energy as fructans, and thus, during periods of stress would draw on these reserves until more favourable conditions return. Due to the similarity in the function of these markers’ BLAST matches, it is proposed that pathways in which energy metabolism and response to stress are controlled in the ARSP plants differ to other varieties, both forage and amenity type. Of the markers that had been assigned consensus map positions, two clusters of markers were observed. Three mapped markers (across 2 contigs) for succinate dehydrogenase grouped ~86cM on LG4, suggesting that these contigs are part of the same gene or two tightly linked gene copies. Haplotype construction from these markers highlighted the unique increased frequency of haplotype “12111” in the 3 Italian ecotypes that were used as founders of the ARSP population, along with S23. This haplotype has been favourably selected in the recurrent breeding programme for increased WSC to now account for >90% of the haplotype frequency. The marker for hexokinase (contig6855) and 2 markers in contig 6714 also grouped together at 35cM on LG1. Turner *et al.*^27^ have previously reported QTL for WSC on LG1 and LG6. These are not the same regions identified in this study and may reflect the polygenic nature of WSC and the high environmental component contributing to this complex trait. As might be expected, LD increases in the ARSP compared to the ecotypes and other varieties due to the recurrent nature of the breeding programme, but remains low compared to inbreeding species such as barley^35^.

Amenity varieties are selected for their suitability for lawns, sports pitches and recreation, requiring a tough sward that thrives on maintenance at a short sward height. This contrasts to forages that need to be highly digestible to animals and have rapid growth yields for grazing or cutting. The contrasting phenotypic traits and the genetic control of these can be dissected by the use of genetic markers. The differentiation between forage and amenities in Fig. 1b can also be used to begin to identify markers that may be associated with traits that differentiate both functional groups (Table 3). However, this is confounded on PC2 by the presence of amenity cultivars bred in Aberystwyth and those of other commercial breeders. There are some striking differences in allele frequencies seen between the OA and AA groups, although the number of accessions included in the OA group greatly outnumbers that of the AA (15 vs 2). As with the ARSP, contributing markers (where mapped) formed two large clusters (~74cM on LG6 and ~46cM on LG7). Two markers contributed a greater weighting to PC2 than others. Pheophorbide A chloroplast-like has been associated with chlorophyll breakdown and senescence in *Arabidopsis*^36^. Prolonging the colour of varieties used for amenity purposes would be more aesthetically desirable to the turf industry^37^,^38^. In contrast, aminoacylase has been associated to *Phytophthora infestans* resistance in *Nicotiana*^39^: it is known that AberImp and AberSprite are susceptible to crown rust infection (D. Thorogood, pers. comm). Two other markers, NAC secondary wall thickening promoting factor 1 and cellulose synthase were also identified in the top 8 markers differentiating OA, AA and OF. This is unsurprising given the differences in cell wall robustness required from forages and amenity varieties. Forages need to be easily digested in contrast to amenities that need to tolerate frequent trampling.

In summary, this study has allowed quantification of genetic variation in ecotypes and cultivars of *L. perenne* and the identification of key regions of the genome that are strongly associated with the differentiation of forage and amenity forms. In addition candidate genes implicated in the control of WSC accumulation have been identified based on a combination of multidimensional and haplotype analysis of genetic variation in ecotypes and commercial breeding germplasm, illustrating the power of crop diversity to both improve nutritional composition and the underlying genetic basis of WSC in ryegrass.

## Methods

### Plant material

Leaf tissue from 249 individuals from 33 accessions of commercial varieties of *Lolium perenne* was collected. These represented 9 accessions from commercial forage varieties (OF) and 7 accessions from Aberystwyth University’s forage breeding programme (AF), in addition to 15 accessions of commercial amenity varieties (OA) and 2 accessions of Aberystwyth amenity varieties (AA) (Supplementary Table 5). DNA was extracted using QIAGEN 96 plant tissue extraction kit.

For a more accurate representation of the effect of a recurrent selection breeding programme on the genetic diversity contained within a cultivar, the AF group was subsetted to Aberystwyth Recurrent Selection Programme (ARSP). The ARSP group contains the 4 most recent forage varieties generated by the programme (AF4-7; Supplementary Table 5) genotyped and recurrently selected with emphasis on increasing WSC. AF3 is also a high sugar variety (Aurora), however, this is not part of the same recurrent selection breeding program as AF4-AF7.

In addition to the cultivars, 716 individual *Lolium perenne* ecotypes as previously described^20^ were also included in this study. Briefly, this included 8 individuals from 90 different geographic locations across Europe (Supplementary Table S1).

In order to generate a robust consensus linkage map, three mapping populations were genotyped. The first population was a back-cross mapping family of 162 individuals was derived from an initial cross between contrasting amenity-type (ex cv AberImp) and forage-type (accession Ba12142 ex Cardigan Island [Wales, UK]) ecotype collection (grid ref 52.1167 -4.6833) genotypes, followed by backcrossing of a single F1 genotype to the amenity type parent^40^. The second population involved 192 F1 progeny from a cross between Aurora and AberMagic^41^. A F2 mapping population of 96 individuals derived from a single hybrid self-pollinating plant obtained from crossing cultivars ‘Perma’ and ‘Aurora’ formed the third mapping population^42^.

### Genotyping

All cultivars (n = 249), ecotypes (n = 716) and 3 mapping populations (n = 450), were genotyped using our custom Illumina Infinium iSelect array across 3425 SNPs. The cluster file trained using the diverse European ecotype panel (as reported and verified in^20^) was applied to the commercial varieties and mapping populations generating genotype calls for 2501 SNPs. Markers with more than 10% missing data (n = 4) and/or a minor allele frequency of less than 5% (n = 225) were excluded. In addition to which, markers which had incorrect heritability in the AberMagic x Aurora mapping population (n = 43) or a probability of less than 0.5 for observed heterozygosity excess in each of the accessions (n = 34, GenePop^43^) were also excluded to minimise technical genotyping errors. In order to verify the technical robustness of the array, the expected number of heterozygotes based on the parental genotypes for the AberMagic x Aurora population were calculated and compared to the observed values. No bias was observed across all markers (Supplementary Table S4). Following these exclusion parameters that had also been applied in Blackmore *et al.*^20^, a final validated marker set of 2199 SNPs spanning 1615 contigs were used for analysis.

### Genetic diversity analysis

Diversity measures were calculated within each of the accessions using GenAlEx^44^ using accession as a population (8 individuals per accession) for the ecotypes and varieties. A summary of values for the varieties was calculated based on the commercial type; amenity or forage. Allele frequency for each marker within an accession was calculated, with up to 8 individuals from each accession used as detailed in Blackmore *et al.*^20^. Principal component analysis (PCA) was performed using the R package. Haplotype frequency was performed using the EM algorithm to estimate maximum likelihood frequencies at the haplotype level in Arlequin 3.5.2.1^45^.

### Construction of a genetic linkage map for Lolium perenne

From the genotyping data of the three individual mapping families, genetic linkage maps were created using JoinMap v4.0^46^. Of the 2199 SNP markers, 1161, 1275 and 691 informative markers were mapped in the Amenity x Forage, AberMagic x Aurora and F2 mapping populations respectively. LOD thresholds of 6.0 and 5.0 were able to separate seven distinctive groups representing the seven chromosomes of *Lolium perenne* in the Amenity x Forage and F2 mapping populations. In the AberMagic x Aurora population LOD threshold of 4.0 was able to separate all but two of the linkage groups. A LOD threshold of 8.0 was required to separate out linkage groups 2 and 4. Groups were assigned to linkage groups that align to those determined for the seven *Hordeum vulgare* chromosomes^47^ and are standardised for all members of the Triticeae family. This was done by BLASTing a small number of contig sequences from markers on each linkage group against *Brachypodium* and rice genomes using the Gramene database^48^ and assigning linkage groups based on comparative genomics of the Triticeae family with these model species (see for example Pfeifer *et al.*^49^). Once assigned to groups, markers with identical genotype scores were excluded and map order was determined using the weighted least squares (linear regression) method^50^ using marker linkages with a recombination threshold of 0.4 and a LOD threshold of 1.0 with map distances calculated from recombination frequencies using Haldane’s mapping function. Three rounds of mapping were employed. An integrated map was then produced by combining the linkage groups of the three maps using the same recombination frequency, LOD score threshold and mapping functions as used for mapping the separate mapping families. Text-based map files were exported to the program MapChart^51^ for production of map images.

### Linkage Disequilibrium

Linkage disequilibrium (LD) was calculated for both the ecotypes and the varieties using PopGen (1.0–3) package in R project and plotted against the genetic map distances between markers as generated from the consensus linkage map. LD was calculated for each linkage group, and then combined to produce genome wide LD plots. A LOWESS curve was included in each plot. This was repeated for the ecotypes, varieties (all cultivars) and ARSP.

## Additional Information

**How to cite this article**: Blackmore, T. *et al.* Germplasm dynamics: the role of ecotypic diversity in shaping the patterns of genetic variation in *Lolium perenne*. *Sci. Rep.* **6**, 22603; doi: 10.1038/srep22603 (2016).

## Supplementary Material

Supplementary Information

Supplementary Tables

## Figures and Tables

**Table 1:** Summary of heterozygosity and F-statistics across different variety groups.

		Ho	He	F	Polymorphic loci (%)	Fis	Fit	Fst
European ecotypes	Mean	0.324	0.291	−0.106	80.28	−0.112	0.135	0.223
	SE	0.005	0.004	0.011	0.99	0.002	0.002	0.001
AA	Mean	0.273	0.255	−0.069	67.39	−0.065	−0.010	0.052
	SE	0.004	0.003	0.005	0.86	0.006	0.006	0.002
OA	Mean	0.314	0.296	−0.060	82.50	−0.061	0.126	0.176
	SE	0.001	0.001	0.002	1.93	0.003	0.003	0.002
AF	Mean	0.328	0.294	−0.113	76.48	−0.109	0.149	0.235
	SE	0.002	0.002	0.003	4.45	0.004	0.005	0.003
(ARSP)	Mean	0.329	0.305	−0.077	79.47	−0.071	0.091	0.154
	SE	0.003	0.002	0.004	2.23	0.005	0.005	0.002
OF	Mean	0.336	0.310	−0.081	85.07	−0.082	0.072	0.142
	SE	0.002	0.001	0.002	1.31	0.004	0.004	0.002

AA–Aberystwyth bred amenities, OA–Other commercial amenities, AF- Aberystwyth bred forages, ARSP–Recurrent selection programme varieties, OF–Other commercial forages. Ho = Observed Heterozygosity = No. of Hets/N, He = Expected Heterozygosity = 1-sum pi^2, F = fixation index = (he- ho)/He = 1- (he/He). Fis = (Mean He-Mean Ho)/Mean He, Fit = (Ht-Mean Ho)/Ht, Fst = (Ht-Mean He)/Ht.

**Table 2:** Summary of AMOVA results between ecotypes or commercial varieties.

Group	No. of individuals	Number of accessions	Among pops (%)	Within pops (%)	PhiPT	P value
Ecotypes	716	90	30	70	0.298	0.001
AA	16	2	8	92	0.078	0.001
OA	119	15	23	77	0.230	0.001
AF	45	7	28	72	0.283	0.001
(ARSP)	32	4	25	75	0.247	0.001
OF	69	9	18	82	0.180	0.001

AA–Aberystwyth bred amenities, OA–Other commercial amenities, AF- Aberystwyth bred forages, ARSP–Recurrent selection programme varieties, OF–Other commercial forages. 999 pairwise populations and permutations. PhiPT = AP/(WP + AP) = AP/TOT where AP = Est. Var. Among Pops, WP = Est. Var. Within Pops.

**Table 3:** Top 20 markers contributing segregation in Fig. 2.

Top 20 markers on PC1 (segregation of AA from OF and OA groups)				
Marker	LG	Position (cM)	PC1	BLAST
3971_540	3	49.378	−0.046	Putative cysteine-rich receptor-like
9820_93	U	U	−0.045	NADH dehydrogenase
6740_1395	4	55.018	−0.045	PP
31101_1669	3	75.576	0.045	Dihydrolipoyl dehydrogenase-like
41898_129	4	86.787	0.044	Succinate dehydrogenase
41898_187	U	U	−0.044	Succinate dehydrogenase
50733_606	4	86.698	0.044	Succinate dehydrogenase
40643_1058	U	U	0.044	PP
6714_511	U	U	0.043	PP
6714_306	1	34.656	−0.043	PP
37377_605	1	44.526	−0.043	Farnesyl pyrophosphate synthetase
17343_359	U	U	0.042	Glyoxysomal fatty acid beta-oxidation multifunctional protein MFP-a
41281_366	U	U	0.042	Glyoxysomal fatty acid beta-oxidation multifunctional protein MFP-a-like
6714_427	1	35.182	0.042	PP
50733_452	U	U	0.042	Succinate dehydrogenase
6740_966	4	54.881	−0.042	PP
50733_371	4	87.261	0.042	Succinate dehydrogenase
6855_1933	1	36.296	−0.041	Hexokinase 5
7244_936	U	U	−0.041	Probable polygalacturonase-like
41366_306	4	105.346	−0.041	Peptidyl-prolyl cis-trans isomerase h
**Top 20 markers on PC2 (segregation of AA from OF and OA groups)**				
**Marker**	**LG**	**Position (cM)**	**PC2**	**BLAST**
31527_789	U	U	−0.057	Pheophorbide a chloroplastic-like
9552_740	7	42.603	0.056	Aminoacylase-1-like
13591_172	U	U	−0.047	Chloroplast genome
47071_67	7	47.583	−0.046	NA
52864_272	U	U	−0.045	Stress Response protein, Nst1
32698_400	6	13.684	0.045	Alcohol dehydrogenase class 3
22583_156	6	74.525	−0.045	Alkaline/neutral invertase
41908_1026	U	U	0.043	Cellulose synthase catalytic subunit 12
43527_131	U	U	0.043	PP
34965_397	4	74.959	−0.041	Cysteine desulfurase chloroplastic-like
34965_538	U	U	0.041	Cysteine desulfurase chloroplastic-like
40640_795	U	U	0.041	1-deoxy-D-xylulose 5-phosphate reductoisomerase
40920_284	7	48.169	−0.041	Trigger factor-like
34965_1344	4	59.347	0.040	Cysteine desulfurase chloroplastic-like
8982_1259	6	62	−0.040	Acetolactate synthase amino acid binding protein
13979_375	U	U	0.040	Cyclin-like f-box
32206_1218	7	23.072	−0.040	Enolase 1-like
53366_177	U	U	−0.040	Cyclin-like f-box
7528_1208	7	43.297	0.039	Auxin response factor 7b
6762_1533	U	U	−0.039	2-oxoglutarate/malate translocator

PP–predicted protein. U–Unmapped. NA- not available.

**Table 4:** Summary of consensus genetic linkage map.

Linkage Group	Number of mapped markers	LG size (cM)	Average distance between markers
1	171	91.918	0.54
2	197	128.305	0.65
3	199	123.870	0.50
4	279	131.229	0.47
5	109	89.227	0.78
6	208	114.036	0.52
7	223	105.950	0.46
Total	1386	784.535	0.54

## References

[b1] Kovach M. J. & McCouch S. R. Leveraging natural diversity: back through the bottleneck. Curr. Opin. Plant Biol. 11, 193–200 (2008). 18313975 10.1016/j.pbi.2007.12.006

[b2] McCouch S. R., McNally K. L., Wang W. & Sackville Hamilton R. Genomics of gene banks: A case study in rice. Am. J. Bot. 99, 407–423 (2012). 22314574 10.3732/ajb.1100385

[b3] Meyer R. S. & Purugganan M. D. Evolution of crop species: genetics of domestication and diversification. Nat. Rev. Genet. 14, 840–852 (2013). 24240513 10.1038/nrg3605

[b4] Wilkins P. W. Breeding perennial ryegrass for agriculture. Euphytica 52, 201–214 (1991).

[b5] Jupp A. P. & Newman E. I. Morphological and anatomical effects of severe drought on the roots of *Lolium perenne* L. New Phytol. 105, 393–402 (1987). 10.1111/j.1469-8137.1987.tb00876.x 33873904

[b6] Roldan-Ruiz I. *et al.* A comparative study of molecular and morphological methods of describing relationships between perennial ryegrass (*Lolium perenne* L.) varieties. Theoret. Appl. Genet. 103, 1138–1150 (2001).

[b7] Thomas H. & James A. R. Freezing tolerance and solute changes in contrasting genotypes of *Lolium perenne* L. acclimated to cold and drought. Ann. Bot. 72, 249–254 (1993).

[b8] Skot L., Sackville Hamilton N. R., Mizen S., Chorlton K. H. & Thomas I. D. Molecular genecology of temperature response in *Lolium perenne*: 2. Association of AFLP markers with ecogeography. Mol. Ecol. 11, 1865–1876 (2002). 12207735 10.1046/j.1365-294x.2002.01568.x

[b9] Dumsday J. L., Smith K. F., Forster J. W. & Jones E. S. SSR-based genetic linkage analysis of resistance to crown rust (*Puccinia coronate* f. sp. *Lolii*) in perennial ryegrass (*Lolium perenne*). Plant Pathology 52, 628–637 (2003).

[b10] Dracatos P. M. *et al.* Molecular characterisation and genetic mapping of candidate genes for qualitative disease resistance in perennial ryegrass (*Lolium perenne* L.). BMC Plant Biol. 9, 62 (2009). 19450286 10.1186/1471-2229-9-62 PMC2694799

[b11] Bugge G. Genetic-variability in a chemical composition of Italian ryegrass ecotypes. Zeitschrift Fur Pflanzenzuchtung-Journal of Plant Breeding 81, 235–240 (1978).

[b12] Culleton N. & Fleming G. A. Mineral composition of ryegrass cultivars. Irish J. Agri. Res. 22, 21–29 (1983).

[b13] Lee M. R. F. *et al.* Production responses from lambs grazed on *Lolium perenne* selected for an elevated water-soluble carbohydrate concentration. Animal Res. 50, 441–449 (2001).

[b14] Wilkins P. W. & Humphreys M. O. Progress in breeding forage grasses for temperate agriculture. J. Agri. Sci. 140, 129–150 (2003).

[b15] Smit H. J., Tas B. M., Taweel H. Z., Tamminga S. & Elgersma A. Effects of perennial ryegrass (*Lolium perenne* L.) cultivars on herbage production, nutritional quality and herbage intake of grazing dairy cows. Grass Forage Sci. 60, 297–309 (2005).

[b16] Lush W. M. & Rogers M. E. Cutting height and the biomass and tiller density of *Lolium perenne* amenity turfs. J. Appl. Ecol. 29, 611–618 (1992).

[b17] Thorogood D. Perennial ryegrass (*Lolium perenne* L.). In: Turfgrass Biology, Genetics and Breeding (eds. Casler M. D. & Duncan R. R. ) pp75–105 (John Wiley and Sons, 2003).

[b18] Sampoux J. P. *et al.* Breeding perennial ryegrass (*Lolium perenne* L.) for turf usage: an assessment of genetic improvements in cultivars released in Europe, 1974–2004. Grass Forage Sci. 68, 33–48 (2012).

[b19] Glab T., Szewczyk W., Dubas E., Kowalik K. & Jezierski T. Anatomical and morphological factors affecting wear tolerance of turfgrass. Scientia Horticulturae 185, 1–13 (2015).

[b20] Blackmore T., Thomas I., McMahon R., Powell W. & Hegarty M. Genetic-geographic correlation revealed across a broad European ecotypic sample of perennial ryegrass (*Lolium perenne*) using array-based SNP genotyping. Theor. Appl. Genet. 128, 1917–1932 (2015). 26093611 10.1007/s00122-015-2556-3 PMC4572065

[b21] Campbell B. M., Thornton P., Zougmoure R., Van Asten P. & Lipper L. Sustainable intensification: What is its role in climate smart agriculture? Curr. Opin. Environ. Sustain. 8, 39–43 (2014).

[b22] Roldan-Ruiz I., Dendauw J., Van Bockstaele E., Depicker A. & De Loose M. AFLP markers reveal high polymorphic rates in ryegrasses (*Lolium* spp.) Mol. Breeding 6, 125–134 (2000).

[b23] Guthridge G. M. *et al.* AFLP analysis of genetic diversity within and between populations of perennial ryegrass (*Lolium perenne* L.) Euphytica 122, 191–201 (2001).

[b24] Forster J. W., Jones E. S., Batley J. & Smith K. F. Molecular marker based genetic analysis of pasture and turf grasses. In Molecular breeding of forage and turf (eds Hopkins A., Wang Z.-Y., Sledge M., Barker R. E. ). pp 197–239 (Kluwer, Dordrecht, 2004).

[b25] Auzanneau J., Huyghe C., Julier B. & Barre P. Linkage disequilibrium in synthetic varieties of perennial ryegrass. Theor. Appl. Genet. 115, 837–847 (2007). 17701396 10.1007/s00122-007-0612-3

[b26] Humphreys M. O. Water-soluble carbohydrates in perennial ryegrass breeding. II. Cultivar and hybrid progeny performance in cut plots. Grass Forage Sci. 44, 237–244 (1989).

[b27] Turner L. B. *et al.* Dissecting the regulation of fructan metabolism in perennial ryegrass (*Lolium perenn*e) with quantitative trait locus mapping. New Phytol. 169, 45–58 (2006). 16390418 10.1111/j.1469-8137.2005.01575.x

[b28] Miller L. A. *et al.* Increased concentration of water-soluble carbohydrate in perennial ryegrass (*Lolium perenne* L.): milk production from late-lactation dairy cows, Grass Forage Sci. 56, 383–394 (2001).

[b29] Fuentes D. *et al.* A deficiency in the flavoprotein of *Arabidopsis* mitochondrial complex II results in elevated photosynthesis and better growth in nitrogen-limited conditions. Plant Physiol. 157, 1114–1127 (2011). 21921116 10.1104/pp.111.183939 PMC3252148

[b30] Jardim-Messeder D. *et al.* Succinate dehydrogenase (mitochondrial complex II) is a source of reactive oxygen species in plants and regulates development and stress responses. New Phytol. 10.1111/nph.13515 (2015). 26082998

[b31] Casano L. M., Martin M. & Sabater B. Hydrogen peroxide mediates the induction of chloroplastic Ndh complex under photooxidative stress in barley. Plant Physiol. 125, 1450–1458 (2001). 11244124 10.1104/pp.125.3.1450 PMC65623

[b32] Voulgaris I., O’Donnell A., Harvey L. M. & McNeil B. Inactivating alternative NADH dehydrogenases: enhancing fungal bioprocesses by improving growth and biomass yield? Sci. Rep. 2, 322 (2012). 22435085 10.1038/srep00322 PMC3308132

[b33] Lothier J., Lasseur B., Prud’homme M.-P. & Morvan-Bertrand A. Hexokinase-dependent sugar signalling represses fructan exohydrolase activity in *Lolium perenne*. *Func*. Plant Biol. 37, 1151–1160 (2010).

[b34] Timm S. *et al.* Mitochondrial dihydrolipoyl dehydrogenase activtity shapes photosynthesis and photorespiration of *Arabidopsis thaliana*. Plant Cell doi: http://dx.doi.org/10.1105/tpc.15.00105 (2015). 10.1105/tpc.15.00105 PMC4531348 26116608

[b35] Caldwell K. S., Russell J., Langridge P. & Powell W. Extreme population-dependent linkage disequilibrium detected in an inbreeding plant species. Hordeum vulgare. Genetics 172, 557–567 (2006). 16219791 10.1534/genetics.104.038489 PMC1456183

[b36] Pruzinska A., Tanner G., Anders I., Roca M. & Hortensteiner S. Chlorophyll breakdown: Pheophorbide *a* oxygenase is a Rieske-type iron-sulfur protein, encoded by the *accelerated cell death 1* gene. Proc. Natl. Acad. Sci. USA 100, 15289–15264 (2003). 10.1073/pnas.2036571100 PMC299977 14657372

[b37] Thorogood D., Bowling P. J. & Jones R. M. Assessment of turf colour change in Lolium perenne L. cultivars and lines. Int. Turfgrass Soc. Res. J. 7, 729–735 (1993).

[b38] Thorogood D. Varietal colour of *Lolium perenne* L. turfgrass and its interaction with environmental conditions. *Plant Var*. Seeds 9, 15–20 (1996).

[b39] Nakano M. *et al.* Silencing of DS2 aminoacylase-like genes confirms basal resistance to *Phytophthora infestans* in *Nicotiana benthamiana*. Plant Sig. Behav. 9, e28004 (2014). 10.4161/psb.28004 PMC4091584 24514749

[b40] Thorogood D. *et al.* A genetic association between leaf elongation rate and flowering time in perennial ryegrass. In: Proceedings of the 30^th^ Eucarpia Fodder Crops Section Meeting 12-16 May, 2013. Quantitative Traits Breeding for Multifunctional Grasslands and Turf (eds Sokolović D., Huyghe C. & Radović J. ) pp267–271 (Springer Science & Business Media, Dordrecht, the Netherlands, 2014).

[b41] Hegarty M. *et al.* Genotyping by RAD sequencing enables mapping of fatty acid composition traits in perennial ryegrass (*Lolium perenne* (L.)). Plant Biotech. J. 11, 572–581 (2013). 10.1111/pbi.12045 23331642

[b42] Armstead I. P., Turner L. B., King I. P., Cairns A. J. & Humphreys M. O. Comparison and integration of genetic maps generated from F_2_ and BC_1_-type mapping populations in perennial ryegrass. Plant Breeding 121, 501–507 (2002).

[b43] Raymond M. & Rousset F. GENEPOP (version 1.2): population genetics software for exact tests and ecumenism. J. Hered. 86, 248–249 (1995).

[b44] Peakall R. & Smouse P. E. GENALEX 6: genetic analysis in Excel. Population genetic software for teaching and research. Mol. Ecol. Notes 6, 288–295 (2006). 10.1093/bioinformatics/bts460 PMC3463245 22820204

[b45] Excoffier L. & Lischer H. E. L. Arlequin suite ver 3.5: A new series of programs to perform population genetics analyses under Linux and Windows. Mol. Ecol. Res. 10, 564–567 (2010). 10.1111/j.1755-0998.2010.02847.x 21565059

[b46] Van Ooijen J. W. JoinMap® 4, Software for the Calculation of Genetic Linkage Maps in Experimental Populations. (Wageningen, the Netherlands: Kyazma B.V., 2006).

[b47] Kleinhofs A. *et al.* A molecular, isozyme and morphological map of the barley (*Hordeum vulgare*) genome. Theoret. Appl. Genet. 86, 705–712 (1993). 24193780 10.1007/BF00222660

[b48] Monaco M. K. *et al.* Gramene 2013: comparative plant genomics resources. Nuc. Acids Res. 42, D1193–1199 (2014). 10.1093/nar/gkt1110 PMC3964986 24217918

[b49] Pfeifer M. *et al.* The perennial ryegrass Genomezipper: targeted use of genome resources for comparative grass genomics. Plant Physiol. 161, 571–582 (2013). 23184232 10.1104/pp.112.207282 PMC3561004

[b50] Stam P. Construction of integrated genetic-linkage maps by means of a new computer package–Joinmap. Plant J. 3, 739–744 (1993).

[b51] Voorrips R. E. MapChart: software for the graphical presentation of linkage maps and QTLs. J. Hered. 93, 77–78 (2002). 12011185 10.1093/jhered/93.1.77

