# Supplementary material for: Germplasm dynamics: the role of ecotypic diversity in shaping the patterns of genetic variation in Lolium perenne
Source: Sci Rep. 2016 Mar 3;6:22603. doi: 10.1038/srep22603 (PMC4776279; doi:10.1038/srep22603)

Germplasm dynamics: the role of ecotypic diversity in shaping the patterns of genetic variation in *Lolium perenne*

T. Blackmore, D. Thorogood, L. Skot, R. McMahon, W. Powell, M. Hegarty*

Supplementary information:

Supplementary Table S1: Geographic location of ecotype sampling sites with genetic diversity values

Supplementary Table S2: Details of Varieties. In subgroup: AA – Aberystwyth bred amenities, OA – Other commercial amenities, AF- Aberystwyth bred forages, ARSP – Aberystwyth Recurrent selection programme varieties, OF – Other commercial forages

Supplementary Table S3: Mapping population marker segregation for heterozygotes

Supplementary Table S4: Commercial varieties with genetic diversity values

Supplementary Table S5: Top 20 markers contributing to the accession segregation of OF and OA groups that segregate on PC2 in Supplementary fig 1. PP – predicted protein, NA – not available. * denote markers that are also in table 3 (top 20 markers segregating AA vs OA)

Supplementary figure S1: Principal component analysis of varieties and ARSP ecotype founders with the exclusions of AA.

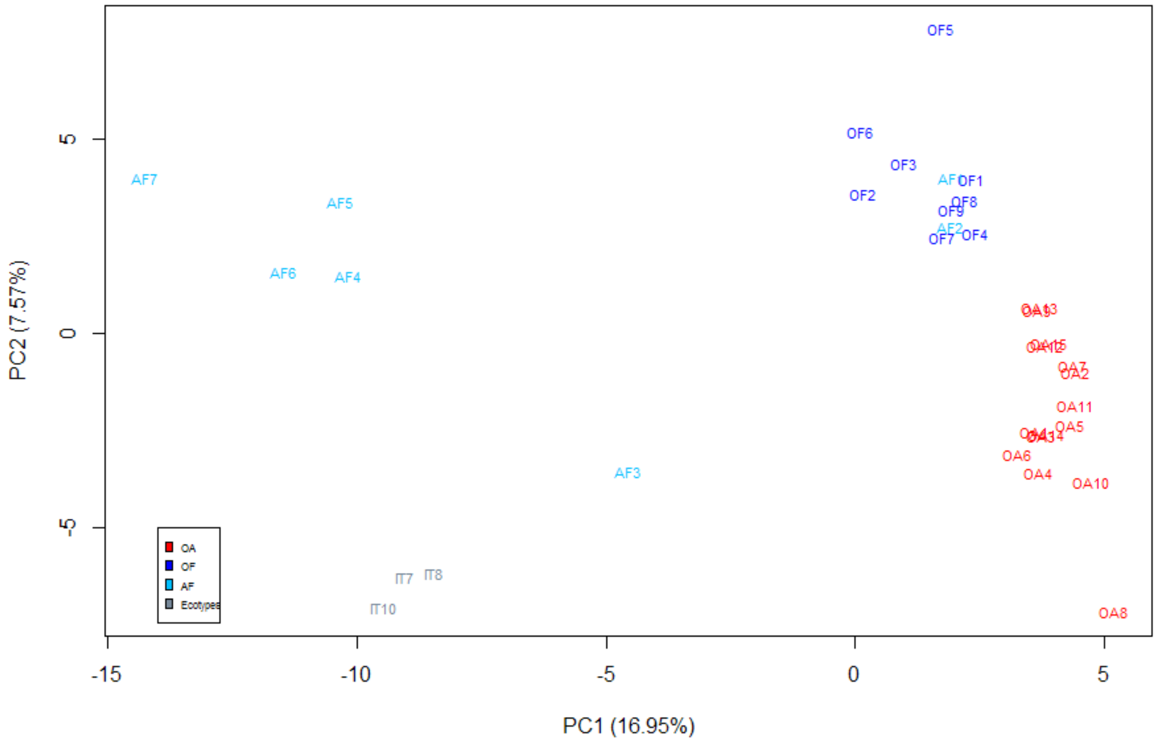

Supplementary figure S2: Consensus *Lolium perenne* genetic linkage map with 1386 SNP markers constructed from 3 mapping populations

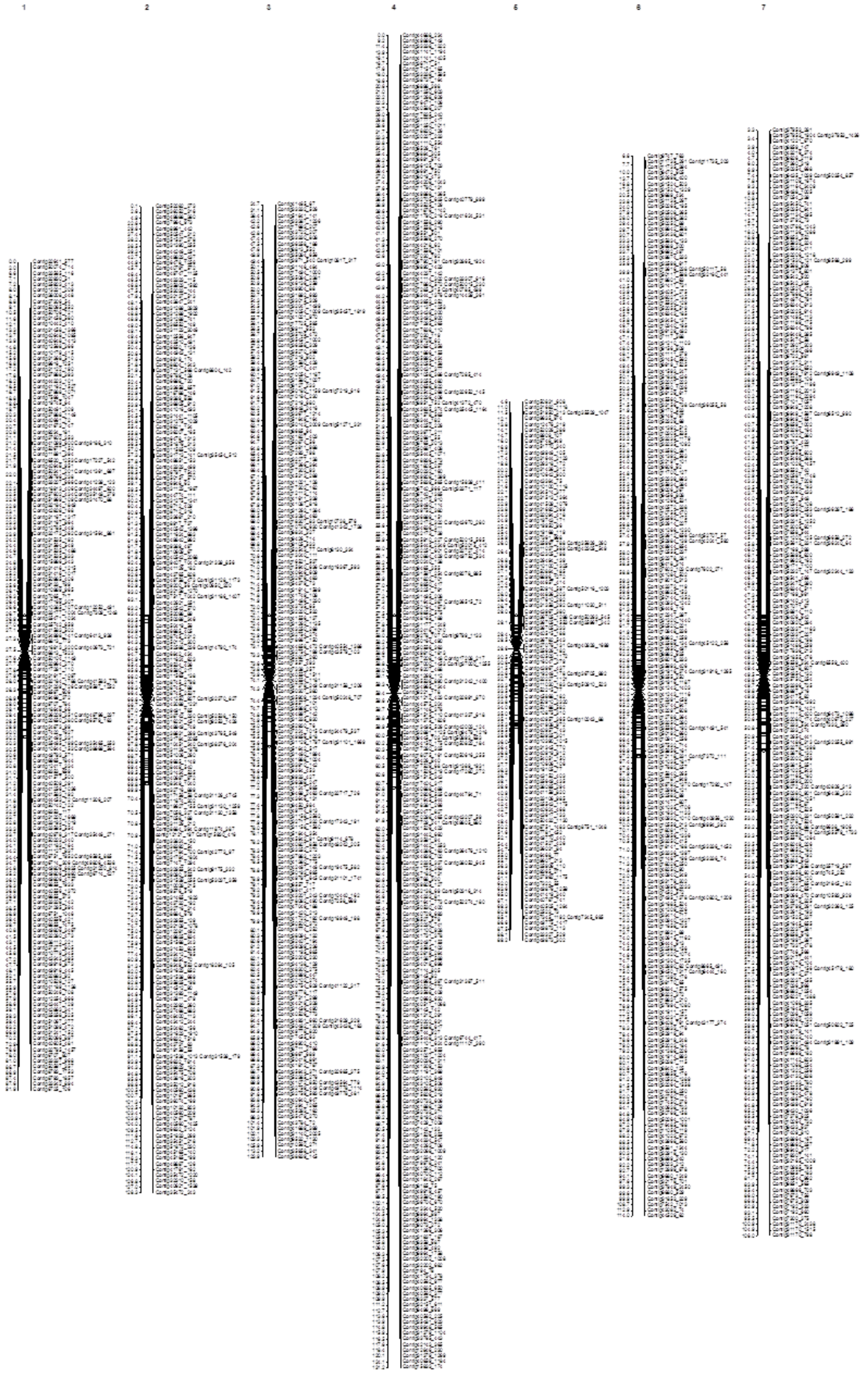

Supplement: Supplementary Information [file srep22603-s1.pdf]
